# The overall survival impact of prophylactic cranial irradiation in limited-stage small-cell lung cancer: A systematic review and meta-analysis

**DOI:** 10.1016/j.ctro.2022.02.002

**Published:** 2022-02-17

**Authors:** Mathijs L. Tomassen, Jacquelien Pomp, Janneke van der Stap, Anne S.R. van Lindert, Max Peters, José S.A. Belderbos, Dirk K.M. De Ruysscher, Steven H. Lin, Joost J.C. Verhoeff, Peter S.N. van Rossum

**Affiliations:** a Department of Radiation Oncology, University Medical Center Utrecht, Utrecht, the Netherlands; b Department of Pulmonology, University Medical Center Utrecht, the Netherlands; c Department of Radiation Oncology, The Netherlands Cancer Institute – Antoni Van Leeuwenhoek Hospital, Amsterdam, the Netherlands; d Department of Radiation Oncology (Maastro), GROW School for Oncology and Developmental Biology, Maastricht University Medical Centre+, Maastricht, the Netherlands; e Department of Radiation Oncology, The University of Texas MD Anderson Cancer Center, Houston (TX), United States of America

## Abstract

**Background:**

Prophylactic cranial irradiation (PCI) for limited-stage small-cell lung cancer (LS-SCLC) patients has become more controversial. Since the publication of the systematic review by Aupérin et al. in 1999, no randomized controlled trials regarding PCI in LS-SCLC have been completed. The aim of this study was to systematically review and meta-analyze the effect of PCI on overall survival (OS) in patients with LS-SCLC.

**Methods:**

A systematic search was conducted in the databases of MEDLINE (PubMed), Embase and the Cochrane library. Only studies that reported an adjusted hazard ratio (aHR), indicating the effect of PCI versus no PCI on OS (adjusted for confounders) in patients with LS-SCLC were included for critical appraisal and meta-analysis. A pooled aHR estimate was calculated using a random-effects model.

**Results:**

Pooling of 28 retrospective studies including a total of 18,575 patients demonstrated a significant beneficial effect of PCI versus no PCI on OS with a pooled aHR of 0.62 (95% CI: 0.57–0.69). Substantial heterogeneity of reported aHRs among studies was observed (I^2^ = 65.9%). Subgroup analyses revealed that this heterogeneity could partly be explained by study sample size. The pooled aHR among 7 versus 21 studies with a sample size of > 300 versus ≤ 300 patients was 0.79 (95% CI: 0.64–0.97) versus 0.56 (95% CI: 0.46–0.69; *p* < 0.001), respectively.

**Conclusions:**

This meta-analysis demonstrates a significant beneficial effect of PCI on OS in patients with LS-SCLC. Larger studies reported a milder beneficial effect, possibly due to a decreased risk of model overfitting. Serious risk of selection and confounding bias were of concern due to the lack of prospective trials. These results support the role of PCI in standard clinical practice in patients with LS-SCLC while awaiting results of prospective trials on alternative strategies.

## Highlights

• PCI for LS-SCLC patients has become more controversial.
• Literature search on PCI impact on overall survival in LS-SCLC yielded 28 studies.
• Meta-analysis of adjusted HRs revealed pooled HR of 0.62 (95% CI: 0.57–0.69).
• Findings support PCI in current practice while awaiting prospective trial results.

## Introduction

Lung cancer is the second most frequent cancer worldwide and the most common cause of cancer-related death [1]. Small cell lung cancer (SCLC) represents about 15% of all lung cancer cases [2]. At diagnosis, 37% of patients is classified as having limited-stage SCLC (LS-SCLC) with no distant metastases (M0) according to the TNM-staging system (8th edition) [3], [4]. Patients with very limited LS-SCLC can be treated with surgery (followed by adjuvant chemotherapy), but the majority of LS-SCLC is treated by concurrent chemoradiotherapy (CRT). If no progression of disease is observed after completion of local and systemic therapy, prophylactic cranial irradiation (PCI) is recommended for the prevention of clinical or radiological manifestation of brain metastases [5].

The *meta*-analysis based on individual patient data of 7 prospective studies (published between 1983 and 1998) conducted by Aupérin et al. still represents the major foundation of international guidelines recommending PCI in LS-SCLC [6]. This *meta*-analysis demonstrated a beneficial effect of PCI on overall survival (OS) in patients with LS-SCLC who had a complete response on a chest X-ray after chemotherapy with or without thoracic radiotherapy. However, limitations of these data in the light of contemporary practice include the use of outdated imaging (e.g. poor resolution CT, unavailability of PET-CT, no or poor brain imaging), patient selection criteria, chemotherapy, supportive care, and radiotherapy techniques in the included studies [6], [7], [8], [9], [10], [11].

PCI for LS-SCLC patients has become more controversial for several reasons, including the lack of new randomized studies since the review of Aupérin et al. [6], the increased quality and availability of brain imaging in contemporary practice, and the increasing knowledge and awareness of neurocognitive side effects of radiotherapy to the brain [12]. After 1999, several non-randomized retrospective studies have reported improved OS after PCI in LS-SCLC [13], [14], [15], but this could not be confirmed by other studies [16], [17], [18]. In addition, a recent randomized study in extensive-stage SCLC (ES-SCLC) without brain metastases suggested equivalence in OS after brain MRI surveillance instead of PCI [19]. In order to overcome current controversies and shortcoming of individual studies, the aim of this study was to perform a systematic review and *meta*-analysis based on published data of the effect of PCI on OS in patients with LS-SCLC.

## Materials and methods

The study protocol was registered in the PROSPERO international database (CRD42021224656, available at http://www.crd.york.ac.uk/prospero). Reporting was performed in accordance with the Preferred Reporting Items for Systematic Reviews and Meta-Analyses (PRISMA) guidelines [20].

## Search strategy

A systematic search was conducted in the databases of MEDLINE (PubMed), Embase and the Cochrane library. The search was last updated November 6, 2021. To identify all studies reporting on the use of PCI in patients with LS-SCLC the terms ‘LS-SCLC’, ‘prophylactic cranial irradiation’ and ‘survival’ in combination were searched, with synonyms and related MeSH terms (Supplementary Table 1).

## Study selection

After deduplication conducted with Mendeley, titles and abstracts were independently screened for eligibility by 3 authors using Rayyan QCRI. Only studies in English, Dutch and German language were included. Studies published before the key systematic review and *meta*-analysis of Aupérin et al. in 1999 were excluded [6]. Any disagreements during the study selection process were solved by reaching consensus. Studies using different types of databases were eligible for inclusion (e.g. SEER database, single-center or multi-center databases). Only studies reporting an adjusted hazard ratio (aHR) with 95% confidence interval (CI), indicating the effect of PCI on OS (adjusted for confounders) in patients with LS-SCLC were included for critical appraisal and *meta*-analysis. The aHR was chosen as primary outcome measure because this represents the least biased within-study estimate of the survival impact of PCI (in contrast to unadjusted HR or crude survival point estimates). Through application of further inclusion criteria (SCLC, PCI) and exclusion criteria (no treatment with chemotherapy, no comparative group of no-PCI, ES-SCLC only, reviews, case-reports or conference abstracts, no full-text available, overlapping publication with the same cohort, or median follow-up < 1 year), the eligibility of the studies was determined by 3 authors independently.

## Data extraction and risk of bias assessment

Data from individual studies was extracted to create an overview of study characteristics (i.e. year of publication, country, study design, primary study determinant, number of patients, age, treatment for primary tumor therapy, PCI dose, use of brain MRI at baseline, and follow-up). By means of the Risk of Bias in Non-randomized Studies or Interventions (ROBINS-I) tool, a risk of bias assessment of the methodological quality was conducted [21]. For each study, 7 domains of bias (i.e. confounding, selection, classification of intervention, deviation from intended intervention, missing data, measurement of outcome, selection of reported results) were graded as having a low, moderate or severe risk of bias. Bias due to confounding was considered as serious if the HRs were adjusted for < 5 parameters. Selection bias was scored as serious when studies unevenly divided partial/complete responders after induction therapy in the PCI group and non-responders in the no-PCI group or if the response to induction therapy was not reported. Two authors performed the risk of bias assessment independently, whereafter consensus was reached.

### Statistical analysis

A *meta*-analysis of the available aHRs indicating the independent association between PCI and OS was conducted using a random-effects model, resulting in a pooled aHR estimate. For determination of heterogeneity among reported aHRs the I^2^ statistic was calculated. An I^2^ between 50 and 90% was considered as substantial heterogeneity in accordance with the Cochrane Handbook for Systematic Reviews [22].

Subgroup analyses were performed with study-level covariates using *meta*-regression random-effects models to study the relation of specific patient-, tumor-, treatment-, and study-related characteristics with the prognostic value of PCI on OS. Cut-off values for subgroups were determined so that each subgroup had a sufficient number of studies. A stratified pooled aHR for each subgroup was calculated. The R^2^ statistic was calculated for each subgroup analysis in order to quantify the amount of overall heterogeneity explained by the subgroup differentiation. Additional *meta*-regression analysis was performed to study potential differences in reported aHRs between studies that performed HR adjustment (versus studies that did not) for age, gender, performance status, tumor size or T-stage, and response to chemotherapy. Analyses were performed using R 4.0.3 software (The R Foundation for Statistical Computing, Vienna, Austria; ‘metafor’ package) and a p-value of < 0.05 was considered statistically significant.

## Results

### Identification of studies

**Fig. 1:**
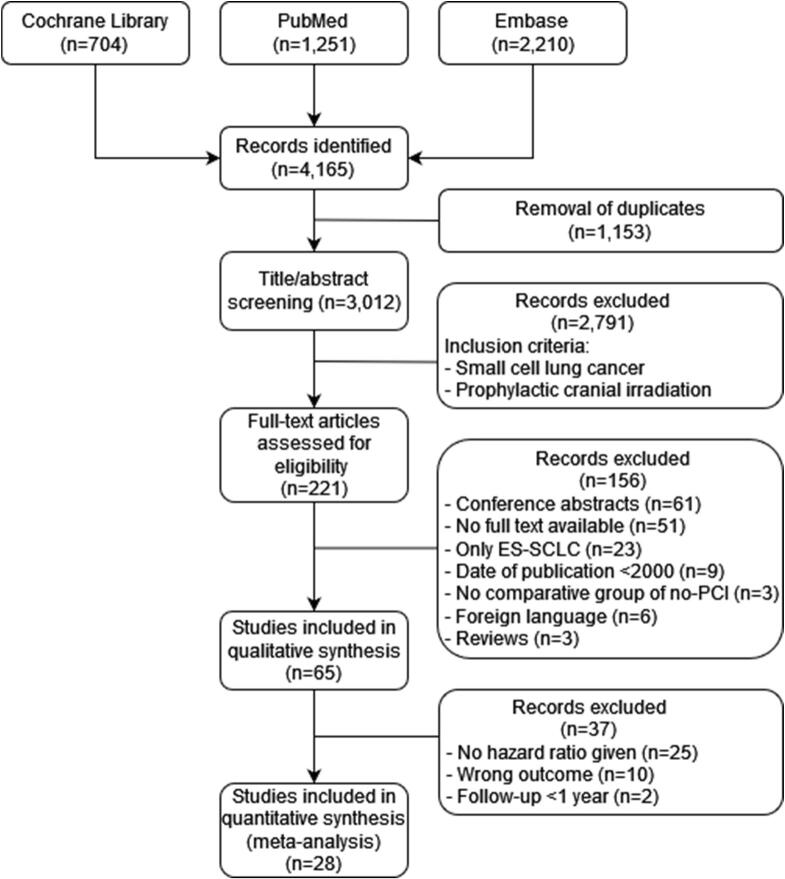
Flowchart summarizing search results and study selection.

A total of 4,165 studies were identified after the systematic search, of which 221 met the inclusion criteria (SCLC, PCI) and these were included for full-text screening. After application of the predefined exclusion criteria, 28 studies including a total of 18,575 patients remained eligible for critical appraisal and *meta*-analysis (Fig. 1).

### Study characteristics

The extracted study characteristics from the 28 included studies are presented in Table 1. Of the 18,575 patients, 3,633 (20%) underwent PCI and 14,942 (80%) were not treated with PCI. All studies were retrospective by design and most were recent with 16 studies (57%) published in or after 2018. Seven studies (25%) had a sample size of > 300 study participants. Mean age of included patients was ≤ 65 years in 15 studies (54%). The majority (64%) of studies originated from Western countries. Twelve (43%) of the studies reported standard acquisition of a brain MRI before considering PCI. The EQD2 (biologically equivalent dose in 2-Gy equivalents) of PCI treatment was > 26 Gy in 6 (21%) of all studies, with the most commonly reported dose regimen being 25 Gy in 10 fractions (46%).

**Table 1:** Study characteristics of studies comparing PCI to no-PCI in patients with limited-stage small-cell lung cancer.

**Study,***year*	Country	Study design	Primary study determinant	PCI (n)	No-PCI (n)	Age (mean)	Primary tumor therapy	PCI dose EQD2 (Gy)	Baseline brain MRI	Median follow-up (months)
**Ng,** 2007 [31]	Australia	Retro	Both	46	44	65	CRT	36	No	50
**Patel,** 2009 [13]	USA	Retro*	PCI	670	7,325	67	NR	NR	NR	13
**Giuliani,** 2010 [24]	Canada	Retro	PCI	127	80	65.7	CRT	26	NR	19
**Bettington,** 2013 [32]	Australia	Retro	TRT	37	42	63.8	CRT	26 or 30 or 36	NR	NR
**Eaton,** 2013 [15]	USA	Retro*	PCI	138	1,788	74.5	CRT	NR	NR	>100
**Zhu,** 2014 [33]	China	Retro	PCI	67	126	56	Surgery	26	Yes	NR
**Xu,** 2016 [34]	China	Retro	PCI	114	234	60	Surgery	NR	NR	NR
**Yang,** 2016 [35]	USA	Retro	PCI	104	850	66.8	Surgery	NR	NR	43
**Eze,** 2017 [36]	Germany	Retro	PCI	71	113	63	CRT	30	Yes	NR
**Farooqi,** 2017 [14]	USA	Retro	PCI	364	294	62	CRT	26	Yes	21
**Wu,** 2017 [37]	USA	Retro	Both	116	167	NR	Both	NR	NR	NR
**Zhang,** 2017 [38]	China	Retro	TRT	94	76	58	CRT	26	NR	30
**Nakamura,** 2018 [39]	Japan	Retro	PCI	93	69	67.5	CRT	26	NR	38
**Sas-Korczynska,** 2018 [40]	Poland	Retro	PCI	167	104	60.5	CRT	30	Yes	33.2
**Yin,** 2018 [41]	China	Retro	PCI	88	52	<60	Both	30 or 32.5	Yes	NR
**Chen,** 2019 [42]	China	Retro	TRT	69	69	<60	Both	26	NR	66
**Kim,** 2019 [43]	South-Korea	Retro	PCI	139	95	61	CRT	26	Yes	22
**Kou,** 2019 [44]	USA	Retro*	PCI	394	2,178	<65	NR	NR	NR	NR
**Resio,** 2019 [45]	USA	Retro	PCI	202	657	66	Surgery	NR	NR	NR
**Elegbede,** 2020 [46]	Canada	Retro	Both	60	60	66	CRT	NR	NR	NR
**Jeong,** 2020 [47]	South-Korea	Retro	TRT	45	56	64	CRT	26	Yes	27
**Lou,** 2020 [48]	China	Retro	PCI	46	100	63	Surgery	NR	Yes	28
**Pezzi,** 2020 [16]	USA	Retro	PCI	84	84	66	CRT	26 or 30	Yes	84
**Ghanta,** 2021 [49]	USA	Retro	PCI	63	50	66	CRT	26	Yes	21.3
**Li,** 2021 [50]	China	Retro	PCI	70	43	<70	CRT	26	Yes	17.8
**Held,** 2021 [51]	Denmark	Retro	PCI	52	27	63.8	CRT	26	Yes	23
**Yan,** 2021 [52]	Canada	Retro	PCI	70	38	65.6	CRT	26	No	22.3
**Zhou,** 2021 [53]	USA	Retro	PCI	43	121	68	Surgery	26	No	NR
CRT: chemoradiotherapy. NR: not reported. PCI: prophylactic cranial irradiation. Retro: retrospective. TRT: thoracic radiotherapy. USA: United States of America. *: SEER database studies.										

PCI was the primary study determinant in 21 studies (75%). In the remaining 7 studies (25%), the association of PCI with OS was reported as secondary outcome. In 17 studies (61%) only patients who underwent CRT for the primary tumor were included, whereas 6 other studies (21%) included surgical patients only, 3 studies (11%) included both surgical and non-surgical patients and 2 studies (7%) lacked reporting on the primary tumor treatment. Among CRT studies, 15 (54% of total) included only patients with complete or partial response to chemotherapy. The median follow-up was > 30 months in 7 studies (25%).

### Quality assessment

An overall moderate to serious risk of bias was observed in the included studies (Table 3). Serious risk of confounding (n = 10, 36%) and selection bias (n = 21, 75%) were observed as a result of the prescription of PCI in patients with partial/complete response to chemotherapy only while including patients with no response to chemotherapy or disease progression in the no-PCI group. Deviation from intended interventions bias was observed in 7 studies (25%) due to poor WHO performance status, patient choice or unknown reasons. None of the included studies reported about missing data. No concerns regarding measurement of outcome bias were found, due to the solid OS outcome.

### Meta-analysis

**Fig. 2:**
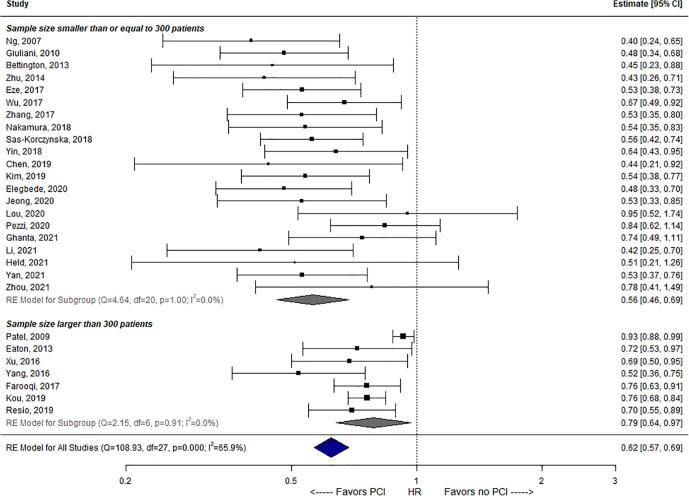
Forest plot of the pooled analysis of 28 studies on the effect of PCI on overall survival in patients with LS-SCLC.

Data on median and 2-year OS estimates are presented in Table 2. Weighted for study sample size, the mean estimate across studies for crude (univariable) median OS was 27.8 versus 18.8 months for PCI versus no-PCI groups. The weighted crude 2-year OS was mean 50.9% versus 26.9% after PCI versus no-PCI. Adjusted HRs of PCI versus no-PCI in LS-SCLC among the 28 studies are presented in Fig. 2. Twenty-three (82%) of 28 studies observed a statistically significant aHR (i.e. 95% CI upper limit < 1) in favor of PCI as opposed to no PCI. Five (18%) of 28 studies observed no significant association between PCI and OS, and no study observed an adverse association between PCI and OS. The pooled aHR across all 28 studies was 0.62 (95% CI: 0.57–0.69). Substantial statistical heterogeneity in aHR estimates among the 28 cohorts was observed (I^2^ = 65.9%).

**Table 2:** Overall survival (OS) outcomes and adjusted hazard ratios of included studies.

**Study**, *year*	Median OS PCI (m)	Median OS No-PCI (m)	2-year OS PCI	2-year OS No-PCI	Adjusted hazard ratio (95% CI)
**Ng,** 2007 [31]	21	14	38%	18%	0.40 (0.24–0.64)
**Patel,** 2009 [13]	24*	20*	42%	23%	0.93 (0.88–0.99)
**Giuliani,** 2010 [24]	23*	10*	20%*	48%*	0.48 (0.33–0.67)
**Bettington,** 2013 [32]	NR	NR	NR	NR	0.45 (0.23–0.88)
**Eaton,** 2013 [15]	20*	16*	33%	12%	0.72 (0.53–0.97)
**Zhu,** 2014 [33]	48*	NRE	93%	63%	0.43 (0.26–0.71)
**Xu,** 2016 [34]	36	26	70%	52%	0.69 (0.50–0.95)
**Yang,** 2016 [35]	NR	NR	NR	NR	0.52 (0.36–0.75)
**Eze,** 2017 [36]	26	14	50%*	10%*	0.53 (0.38–0.73)
**Farooqi,** 2017 [14]	28*	22*	63%*	47%*	0.76 (0.63–0.91)
**Wu,** 2017 [37]	NR	NR	NR	NR	0.67 (0.49–0.92)
**Zhang,** 2017 [38]	32	23	70%	46%	0.53 (0.35–0.80)
**Nakamura,** 2018 [39]	32*	18*	36%	16%	0.54 (0.36–0.82)
**Sas-Korczynska,** 2018 [40]	26	15	52%	30%	0.56 (0.42–0.74)
**Yin,** 2018 [41]	NR	NR	40%	25%	0.64 (0.43–0.95)
**Chen,** 2019 [42]	NR	NR	NR	NR	0.44 (0.22–0.97)
**Kim,** 2019 [43]	31*	16*	59%	36%	0.54 (0.38–0.77)
**Kou,** 2019 [44]	20*	14*	40%*	23%*	0.76 (0.69–0.85)
**Resio,** 2019 [45]	NRE	60	60%	82%	0.70 (0.55–0.89)
**Elegbede,** 2020 [46]	NR	NR	NR	NR	0.48 (0.33–0.70)
**Jeong,** 2020 [47]	NR	NR	NR	NR	0.53 (0.33–0.84)
**Lou,** 2020 [48]	46	49	74%	78%	0.95 (0.52–1.75)
**Pezzi,** 2020 [16]	27	25	60%	58%	0.84 (0.60–1.11)
**Ghanta,** 2021 [49]	36*	24*	63%*	50%*	0.74 (0.49–1.11)
**Li,** 2021 [50]	36*	20*	70%*	43%*	0.42 (0.25–0.70)
**Held,** 2021 [51]	55	24	52%*	27%*	0.51 (0.21–1.28)
**Yan,** 2021 [52]	36*	23*	70%*	38%*	0,53 (0.37–0.76)
**Zhou,** 2021 [53]	76	36	NR	NR	0.78 (0.41–1.49)
**Unweighted median**	31.5	21.0	59.0%	38.0%	–
**Weighted mean**	27.8	18.8	50.9%	26.9%	–
*: Extracted from Kaplan-Meier curve. m: months. NR: not reported. NRE: not reached.

### Subgroup analyses

Results from study-level subgroup analyses are presented in Table 4. A statistically significant difference in pooled aHR estimates was found for 21 studies with a sample size of ≤ 300 patients versus 7 studies with > 300 patients (i.e. pooled aHR 0.56 versus 0.79, respectively, *p* < 0.001; Fig. 2). This subgroup stratification accounted for 60.7% of the overall heterogeneity (R^2^). Between other subgroups of studies (i.e. based on publication year, mean age, brain MRI at baseline, total radiation dose, primary study determinant, treatment for primary tumor, response to chemotherapy, median follow-up), no statistically significant differences in pooled aHRs were identified.

**Table 3:** ROBINS-I risk of bias assessment.

**Table 4:** Results from study-level subgroup analyses for prognostic value of PCI versus no PCI on overall survival.

Factor	n^†^	Stratified HR (95% CI)	*p* value	I^2^	R^2^
*Publication year:*			0.989	61.5%	0.0%
Before 2018	12	0.66 (0.53–0.82)			
In or after 2018	16	0.63 (0.52–0.78)			
*Sample size:*			<0.001*	41.0%	60.7%
≤300 patients	21	0.56 (0.46–0.69)			
>300 patients	7	0.79 (0.64–0.97)			
*Mean age:*			0.432	61.5%	0.0%
≤65 years	15	0.61 (0.50–0.76)			
>65 years	12	0.69 (0.55–0.86)			
*Country of origin:*			0.154	62.5%	12.3%
Eastern	10	0.56 (0.42–0.75)			
Western	18	0.69 (0.59–0.82)			
*Brain MRI at baseline:*			0.906	65.3%	0.0%
No or not reported	16	0.67 (0.56–0.81)			
Yes	12	0.62 (0.48–0.80)			
*Total radiation dose:*				56.2%	26.5%
26 Gy (EQD2_α/β=10_)	13	0.57 (0.44–0.73)	*Ref*		
>26 Gy (EQD2_α/β=10_)	6	0.58 (0.41–0.82)	0.801		
Not reported	9	0.77 (0.62–0.94)	0.020*		
*Primary study determinant:*				63.0%	13.8%
PCI	21	0.69 (0.59–0.81)	*Ref*		
Thoracic radiotherapy	4	0.50 (0.30–0.83)	0.110		
Both	3	0.52 (0.32–0.86)	0.165		
*Treatment for primary tumor:*				49.9%	38.9%
Chemoradiotherapy	17	0.58 (0.47–0.71)	*Ref*		
Surgery	6	0.65 (0.45–0.93)	0.364		
Both or not reported	5	0.80 (0.63–1.02)	0.008*		
*Response to chemotherapy:*			0.151	60.9%	10.9%
Only complete or partial	15	0.58 (0.47–0.73)			
Not reported	13	0.72 (0.60–0.88)			
*Median follow-up:*				60.3%	0.0%
≤30 months	11	0.69 (0.54–0.87)	*Ref*		
>30 months	7	0.59 (0.42–0.81)	0.522		
Not reported	10	0.64 (0.50–0.82)	0.818		

HR: hazard ratio. USA: United States of America. *p* value significance of difference between stratified HR as compared to reference (*Ref*) subgroup. I^2^: residual heterogeneity/unaccounted variability in the *meta*-regression model. R^2^: amount of heterogeneity accounted for by including the factor in the *meta*-regression model. 95% CI: 95% confidence interval. ^†^: number of studies.

Results from study-level subgroup analyses with respect to HR adjustments are presented in Supplementary Table 2. Reported aHRs among 15 studies that adjusted for tumor size or T-stage were significantly higher in comparison with the aHRs among 12 studies that lacked adjustment for tumor size or T-stage (pooled aHR 0.74 versus 0.54, respectively, *p* = 0.002). In the other studied subgroups based on the type of HR adjustment no significant difference between the pooled aHRs was observed.

## Discussion

In many countries including the USA and The Netherlands a significant declining trend of PCI administration over the past decade has been reported not only in ES-SCLC, but also in LS-SCLC patients [12], [23]. Level 1b randomized clinical trial data has likely been an explanation for the decreased use of PCI in ES-SCLC with MRI surveillance as alternative [19]. Over the past 25 years, no such prospective data was published on the impact of PCI in LS-SCLC. The current *meta*-analysis based on 28 retrospective studies demonstrated a pooled adjusted HR of PCI versus no PCI for OS of 0.62 (95% CI: 0.57–0.69). Importantly, none of these available studies were randomized or prospective by design. However, since even studies in more recent years support this apparent beneficial effect of PCI on OS for patients with LS-SCLC PCI remains an important standard treatment modality when the aim is to prolong survival.

Subgroup analysis demonstrated that aHR estimates for PCI have not significantly changed in more recent years (i.e. since 2018 compared to before 2018). This could be related to the fact that the techniques to plan and deliver PCI has not substantially changed over the last decades. Rather, two explanations for the observed heterogeneity in HR estimates among studies were revealed statistically. First, when stratifying studies with a sample size > 300 patients versus ≤ 300 patients, treatment with PCI in studies with > 300 patients appeared somewhat less (but still significantly) associated with favorable OS in LS-SCLC when compared to studies with ≤ 300 patients (pooled aHR 0.79 versus 0.56, *p* < 0.001). A possible explanation could be that smaller sample sizes more likely resulted in overoptimism of the effect of PCI due to a higher chance of model-overfitting (i.e. adding too many variables in the multivariable model) compared to studies with a larger sample size.

The apparent survival advantage of PCI in LS-SCLC is thought to arise from preventing or delaying manifestation of brain metastases, as supported by reported 3-year incidence rates of brain metastasis decreasing from 53% to 23% [24]. This advantageous effect of PCI must be weighed against its disadvantages. The key EORTC trial conducted by Slotman et al. demonstrated a negative acute effect on health-related quality of life in the first 3 months after PCI, mainly due to fatigue and hair loss [25]. In addition, among others the phase II RTOG 0212 trial that compared different total doses of PCI demonstrated that PCI is associated with late adverse events such as chronic neurotoxicity (60% after 12 months) and neurologic deterioration (62% after 12 months) [26]. However, that trial had no comparative group of patients with no-PCI.

Alternative approaches to conventional PCI have been proposed. First, in a randomized phase III trial in ES-SCLC MRI surveillance instead of PCI (with MRI surveillance as well) has been shown to result in comparable overall survival with a potential increased sparing of neurocognitive functioning [19]. Importantly, in that trial 83% of patients in the MRI surveillance group still required radiotherapy to the brain due to detection of brain metastases during follow-up [19]. However, no such trial has been completed in LS-SCLC. Second, PCI with hippocampal avoidance (HA-PCI) is a new treatment option to reduce neurocognitive side effects. A Dutch multicenter randomized phase III trial NCT01780675 (including 168 patients) did not reveal a lower probability of cognitive decline (measured by total recall on the revised Hopkins Verbal Learning Test) in patients with SCLC treated with HA-PCI versus conventional PCI [27]. However, the randomized phase III PREMER trial (including 150 SCLC patients) did demonstrate a beneficial effect on cognitive function for HA-PCI using the delayed free recall, free and cued selective reminding, and total recall [28]. Therefore, the role of HA-PCI has not been sufficiently demonstrated and results of ongoing trials like the phase III NRG CC003 trial evaluating HA-PCI are to be awaited.

The results of this *meta*-analysis were merely based on retrospective comparative data, which stresses the importance of prospective randomized trials. The ongoing phase III MAVERICK trial that started in 2020 investigates the effect of MRI surveillance alone versus MRI surveillance with PCI on OS in both ES-SCLC and LS-SCLC patients. This trial aims to include 668 participants and besides OS as primary objective, also brain metastasis-free survival, cognitive failure-free survival and toxicities will be investigated [29]. In addition, EORTC recently initiated the phase III PRIMALung trial, in which 600 patients with either ES-SCLC and LS-SCLC will be randomized to MRI surveillance versus MRI surveillance plus PCI [30]. The primary endpoint is OS and secondary endpoints include cognitive failure-free survival, quality of life, and safety profiling of PCI.

This systematic review and *meta*-analysis has several limitations inherent to drawbacks of the included studies. Firstly, all included studies had a retrospective design causing confounding and selection bias. In calculating aHRs in multivariable survival models, studies attempted to minimize confounding bias but several studies only adjusted for a small number of confounders. Secondly, a large variety in patient selection across the studies was observed in terms of what response to chemotherapy was allowed (not reported/complete/partial/no response). Imbalances mostly due to a larger number of patients with no response to chemotherapy in the no-PCI group, may have partly biased the HR estimates falsely disfavoring no-PCI. Thirdly, publication bias could be present in literature, for example because investigators (and reviewers) would not expect outcomes of PCI to be different from the *meta*-analysis conducted by Aupérin et al [6]. However, this *meta*-analysis was strengthened by the large amount of studies (n = 28), patient numbers (n = 18,575) and small residual (unexplained) heterogeneity after *meta*-regression analyses.

In conclusion, this *meta*-analysis of 28 studies demonstrated that patients with LS-SCLC who underwent PCI had a 38% decreased risk of death over time compared to patients who did not receive PCI. These results support the effective role of PCI in standard clinical practice in patients with LS-SCLC with no progression after systemic treatment, and underline the need for (ongoing) prospective trials before considering alternative strategies.

**Funding statement**

No external funding was involved in this study.

**Data availability statement**

All data generated and analyzed during this study are included in this published article (and its Supplementary information files).

## Declaration of Competing Interest

The authors declare that they have no known competing financial interests or personal relationships that could have appeared to influence the work reported in this paper.
