# Supplementary material for: The overall survival impact of prophylactic cranial irradiation in limited-stage small-cell lung cancer: A systematic review and meta-analysis
Source: Clin Transl Radiat Oncol. 2022 Feb 17;33:145–52. doi: 10.1016/j.ctro.2022.02.002 (PMC8881197; doi:10.1016/j.ctro.2022.02.002)
Supplement: Supplementary Data 1 [file mmc1.docx]

| Supplementary Table 1. Search strategy and results as on 6 November 2021. | | |
| --- | --- | --- |
| Database | **Search query** | **Results** |
| Pubmed | (("small cell lung"[Title/Abstract] OR sclc[Title/Abstract] OR "oat cell lung"[Title/Abstract] OR "Small Cell Lung Carcinoma"[Mesh]) AND ((prophylactic cranial[Title/Abstract] OR pci[Title/Abstract] OR prophylactic brain[Title/Abstract] OR prophylactic cerebral[Title/Abstract] OR whole brain radiation[Title/Abstract] OR whole brain irradiation[Title/Abstract] OR wbrt[Title/Abstract] OR whole brain radiotherapy[Title/Abstract]) OR ("Cranial Irradiation"[Mesh]))) AND (overall survival[Title/Abstract] OR mortality[Title/Abstract] OR surviv*[Title/Abstract]) | 1,251 |
| Embase | ((small AND ('cell'/exp OR cell) AND lung:ti,ab,kw OR sclc:ti,ab,kw OR 'oat'/exp OR oat) AND ('cell'/exp OR cell) AND lung:ti,ab,kw OR small) AND ('cell'/exp OR cell) AND ('lung'/exp OR lung) AND ('carcinoma'/exp OR carcinoma)  AND ((((((prophylactic AND cranial:ti,ab,kw OR pci:ti,ab,kw OR prophylactic) AND brain:ti,ab,kw OR prophylactic) AND cerebral:ti,ab,kw OR whole) AND ('brain'/exp OR brain) AND radiation:ti,ab,kw OR whole) AND ('brain'/exp OR brain) AND irradiation:ti,ab,kw OR wbrt:ti,ab,kw OR whole) AND ('brain'/exp OR brain) AND radiotherapy:ti,ab,kw OR 'cranial irradiation'/exp OR 'cranial irradiation')  AND (overall AND survival:ti,ab,kw OR mortality:ti,ab,kw OR surviv*:ti,ab,kw) | 2,210 |
| Cochrane | #1 small cell lung:ti,ab,kw OR SCLC:ti,ab,kw OR oat cell lung:ti,ab,kw OR small cell lung carcinoma:ti,ab,kw  #2 prophylactic cranial:ti,ab,kw OR pci:ti,ab,kw OR prophylactic brain:ti,ab,kw OR prophylactic cerebral:ti,ab,kw OR whole brain ardiation:ti,ab,kw OR whole brain irradiation:ti,ab,kw OR wbrt:ti,ab,kw OR whole brain radiotherapy:ti,ab,kw OR cranial irradiation:ti,ab,kw  #3 overall survival:ti,ab,kw OR mortality:ti,ab,kw OR survival:ti,ab,kw  #4 #1 AND #2 AND #3  #5 MeSH descriptor: [Small Cell Lung Carcinoma] explode all trees  #6 MeSH descriptor: [Small Cell Lung Cancer] explode all trees  #7 (#1 OR #5) AND (#2 OR #6) AND #3 | 704 |
|  |  | **4,165** |

| **Supplementary Table 2.** Results from study-level subgroup analyses regarding HR adjustments for prognostic value of PCI versus no PCI on overall survival. | | | | | |
| --- | --- | --- | --- | --- | --- |
| **HR adjustment for:** | **n^†^** | **Stratified HR (95% CI)** | ***p* value** | **I^2^** | **R^2^** |
| *Age:* |  |  | 0.534 | 57.4% | 0.0% |
| No | 7 | 0.71 (0.54-0.93) |  |  |  |
| Yes | 20 | 0.63 (0.52-0.76) |  |  |  |
|  |  |  |  |  |  |
| *Gender:* |  |  | 0.084 | 58.7% | 19.2% |
| No | 9 | 0.57 (0.43-0.75) |  |  |  |
| Yes | 18 | 0.71 (0.60-0.84) |  |  |  |
|  |  |  |  |  |  |
| *Performance status:* |  |  | 0.609 | 64.0% | 0.0% |
| No | 11 | 0.69 (0.54-0.87) |  |  |  |
| Yes | 11 | 0.61 (0.47-0.79) |  |  |  |
|  |  |  |  |  |  |
| *Tumor size or T-stage:* |  |  | 0.002* | 47.9% | 48.0% |
| No | 12 | 0.54 (0.42-0.70) |  |  |  |
| Yes | 15 | 0.74 (0.62-0.88) |  |  |  |
|  |  |  |  |  |  |
| *Response to chemotherapy:* |  |  | 0.956 | 65.6% | 0.0% |
| No | 21 | 0.67 (0.57-0.79) |  |  |  |
| Yes | 6 | 0.63 (0.45-0.89) |  |  |  |
|  |  |  |  |  |  |
| HR: hazard ratio. *p* value: significance of difference between stratified HR as compared to reference subgroup. I^2^: residual heterogeneity / unaccounted variability in the meta-regression model. R^2^: amount of heterogeneity accounted for by including the factor in the meta-regression model. 95% CI: 95% confidence interval. ^†^: number of studies. | | | | | |
